# A Nomogram With Six Variables Is Useful to Predict the Risk of Acquiring Carbapenem-Resistant Microorganism Infection in ICU Patients

**DOI:** 10.3389/fcimb.2022.852761

**Published:** 2022-03-25

**Authors:** Jin Zhang, Wanjun Liu, Wei Shi, Xuanxuan Cui, Yu Liu, Zongqing Lu, Wenyan Xiao, Tianfeng Hua, Min Yang

**Affiliations:** ^1^ The 2nd Department of Intensive Care Unit, The Second Affiliated Hospital of Anhui Medical University, Hefei, China; ^2^ The Laboratory of Cardiopulmonary Resuscitation and Critical Care Medicine, The Second Affiliated Hospital of Anhui Medical University, Hefei, China; ^3^ Key Laboratory of Intelligent Computing and Signal Processing, Ministry of Education, Anhui University, Hefei, China

**Keywords:** LASSO regression, nomogram, MIMIC-IV database, intensive care unit, carbapenem-resistant microorganisms

## Abstract

**Background:**

Carbapenem-resistant microorganism (CRO) transmission in the medical setting confers a global threat to public health. However, there is no established risk prediction model for infection due to CRO in ICU patients. This study aimed to develop a nomogram to predict the risk of acquiring CRO infection in patients with the first ICU admission and to determine the length of ICU stay (ICU-LOS) and 28-day survival.

**Methods:**

Patient data were retrieved from the Medical Information Mart for Intensive Care (MIMIC-IV) database based on predetermined inclusion and exclusion criteria. A CRO was defined as a bacterium isolated from any humoral microbial culture that showed insensitivity or resistance to carbapenems. The characteristics of CRO and non-CRO patients in the first ICU admission were compared. Propensity score matching was applied to balance the differences between the CRO and non-CRO cohorts. Kaplan–Meier curves were constructed to determine the 28-day survival rate and ICU-LOS. Furthermore, after randomization of the CRO cohort into the training and validation sets, a predictive nomogram was constructed based on LASSO regression and Logistic regression analysis, and its performance was verified by internal validation.

**Results:**

Overall, 4531 patients who had first ICU admission as recorded in MIMIC-IV were enrolled, 183 (4.04%) of whom were diagnosed with CRO infection. Moreover, CRO infection was independently associated with 28-day survival and ICU-LOS in ICU patients. Parameters eligible for inclusion in this nomogram were male sex, hemoglobin-min, temperature-max, use of a peripherally inserted central catheter line, dialysis treatment, and use of carbapenems. This nomogram showed a better performance as indicated by the area under the receiver operating characteristic curve values of 0.776 (95% confidence interval [CI] 0.667-0.750) and 0.723 (95% CI 0.556-0.855) in the training and validation sets, respectively, in terms of predicting the risk of acquiring CRO infection.

**Conclusions:**

CRO infection was independently associated with ICU-LOS and 28-day survival in patients with first ICU admission. The nomogram showed the best prediction of the risk of acquiring CRO infection in ICU patients. Based on the nomogram-based scoring, we can management the risk factors and guide individualized prevention and control of CRO.

## Introduction

Multidrug-resistant organisms refer to bacteria that present resistance to three or more classes of antimicrobial agents simultaneously; among them, carbapenem-resistant microorganisms (CRO), especially carbapenem-resistant gram-negative bacilli, are the most frequently implicated, including carbapenem-resistant Enterobacteriaceae (CRE), carbapenem-resistant *Acinetobacter baumannii* (CRAB), and carbapenem-resistant *Pseudomonas aeruginosa* (CRPA) (Magiorakos et al., 2012).

Since the past decade, the incidence of CRO infection has been increasing worldwide and has led to a poor prognosis (Xu et al., 2017; Touat et al., 2019; Merrick et al., 2021). CRO infection has also created challenging situations in China. The 2020 national antimicrobial surveillance report suggests that the carbapenem resistance rates of *Klebsiella pneumoniae*, *Pseudomonas aeruginosa*, and *Acinetobacter baumannii* are above 23%, 19%, and 72%, respectively (Hu et al., 2021). Considering the serious global situation, both the World Health Organization and the United States Centers for Disease Control and Prevention have established the CRO risk level as the top rank and have suggested the implementation of active and effective prevention and control measures for CRO infection to ensure patient safety (Guh et al., 2014; WHO Guidelines, 2017).

The emergence and spread of CRO resistance are associated with multiple factors such as inappropriate use of antimicrobials, poor practice of infection prevention and control, and so on (Bonomo et al., 2018). The principle of prevention and control is to take bundling measures to block the spread of CRO (Guh et al., 2014; Wilson et al., 2016; Friedman et al., 2017; Magiorakos et al., 2017; WHO Guidelines, 2017; Solter et al., 2018), the primary measures being strict management of the use of antimicrobials and active screening for infection (Wilson, 2016). The formulation of concrete prevention and control strategies relies on the outcomes of screening (Orsi et al., 2011; Richter and Marchaim, 2017). However, screening is time consuming and costly, and the ideal target population for screening and frequency of testing continue to remain debatable (Birgand et al., 2016; Al-Zahrani, 2018; Kois et al., 2020; Bedos et al., 2021; Merrick et al., 2021).

Recently, as a visualization tool, a nomogram based on multiple logistic regression was developed to predict the risk of CRE infection among liver transplantation recipients (Giannella et al., 2021) and patients in the intensive care unit (ICU) of a secondary referral hospital (Seo et al., 2021). However, there is no established risk prediction model for CRO infection among ICU patients. This study therefore aimed to develop a nomogram to determine the risk of acquiring CRO infection in patients with first ICU admission and to explore the length of ICU stay (ICU-LOS) and 28-day survival in patients with CRO infection.

## Materials and Methods

### Patients and Definitions

This was a retrospective cohort study based on the Medical Information Mart for Intensive Care (MIMIC-IV; version 1.0) database, which contains the data of 53,130 ICU patients of Beth Israel Deaconess Medical Center. The majority of high-quality data in MIMIC-IV are from the electronic health records and an ICU-specific clinical information system. Our record ID of permission to use the database is 39691989. Because the patient privacy information was encrypted in the database, the ethics committee at the medical center did not require informed consent.

**Figure 1:**
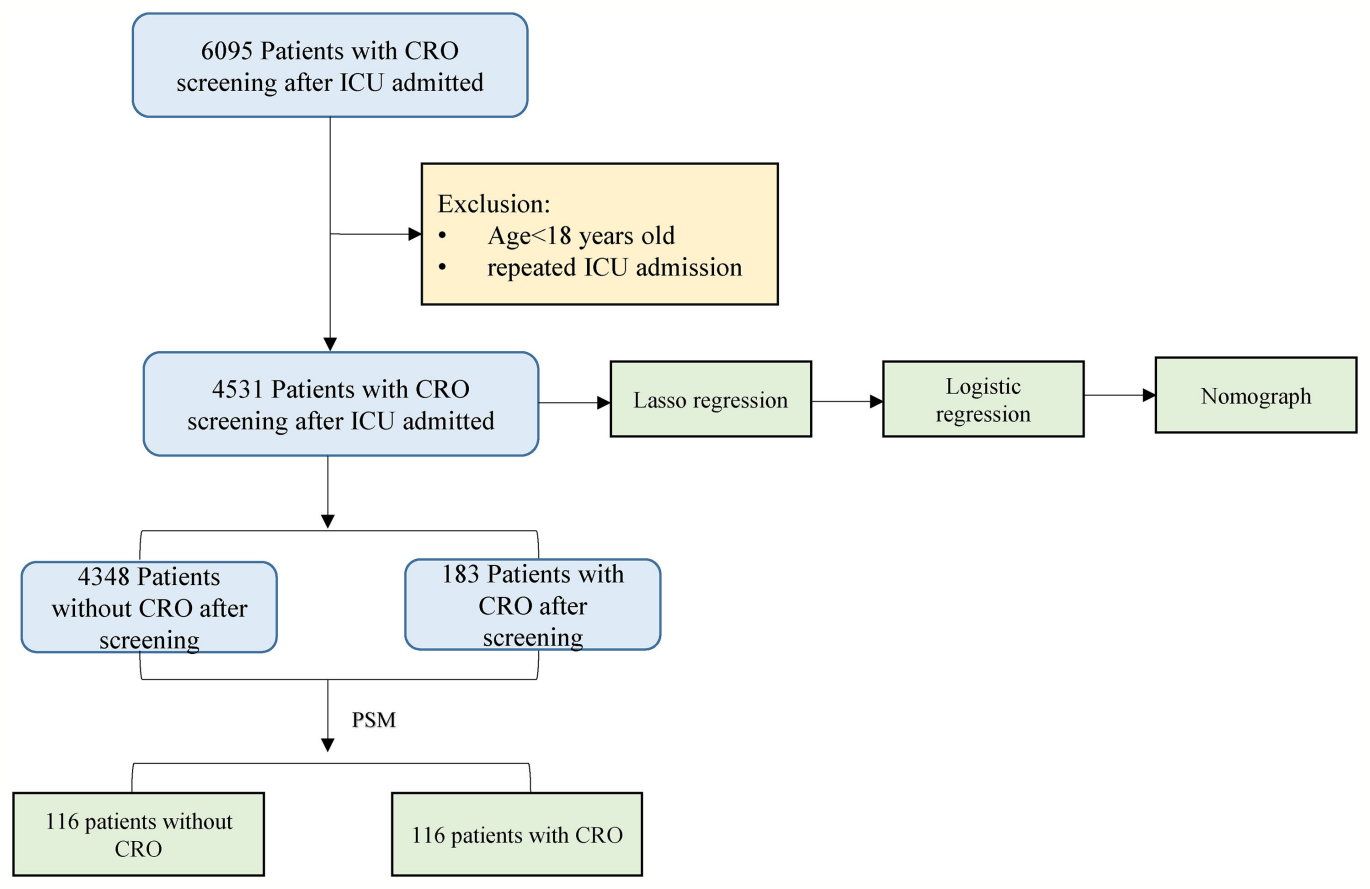
Flowchart of study participants.

In this study, CRO was defined as a bacterium isolated from any humoral microbial culture that showed insensitivity or resistance to carbapenems (Nordmann and Poirel, 2019). The exclusion criteria were as follows: (a) patient age <18 years; (b) repeated ICU admissions; and (c) patients with CRO infection within 48 hours after ICU admission (**Figure 1**).

### Data and Variables

The indexes we extracted were demographics (number of males, age, and weight), basic vital signs (mean arterial pressure, body temperature, heart rate, respiratory rate, peripheral oxygen saturation), laboratory indexes (white blood cell count, platelet count, hemoglobin, blood glucose, blood urea nitrogen, creatinine, prothrombin time, and partial prothrombin time), comprehensive score (Simple Acute Physiology Score II [SAPS II], Sequential Organ Failure Assessment [SOFA] score), comorbidities, invasive catheter use, and therapeutics before culture (renal replacement therapy [RRT], bronchoscopy, use of antibiotics, and chemotherapy). For indicators with multiple measurements, we included only the worst value on the first day of ICU, and the time of therapeutic index was considered from admission to microbial culture.

### Statistical Analysis

Continuous variables are described as median and interquartile range. The Mann–Whitney U-test was used for statistical comparison between two groups. Categorical variables are described as total number and percentage, and the chi-square test or Fisher’s exact test was used for comparison between groups. Propensity score matching (PSM) was used to balance the differences between the CRO and non-CRO groups. We used inverse probability weighting to further adjust for possible imbalances between the variables of the two groups. The Kaplan–Meier (K-M) curves were constructed to determine the 28-day survival rate and compare the data between the two groups, and the differences in survival rates between the two groups were compared using the log-rank test. More than 40% of the missing variables were excluded from the analysis. The remaining missing values were interpolated by multiple interpolation (**Figure S1**).

After randomization of the CRO cohort, all data were divided into the training and test sets in a ratio of 8:2. LASSO regression was used to reduce data dimensions. Then, the data were modeled using a logistic regression model (LRM) after dimensionality reduction. The prediction efficiency of the models was compared using the area under the receiver operating characteristic (AUROC) curve. Additionally, the positive predictive value and the negative predictive value were further determined to evaluate the performance of the model. Structured query language was used to extract the data from the MIMIC-IV database. All statistical analyses were performed using R, version 3.6.2, and statistical significance was set at P < 0.05.

## Results

### Characteristics of the Included Patients With CRO Infection

Overall, 4531 patients were enrolled in this study, 4.04% of whom were patients with CRO infection. **Table 1** shows the characteristics of all included patients on ICU admission. Interestingly, we found that there was no significant difference between the CRO group and the non-CRO group in terms of comorbidities. However, the proportion of male was greater in the CRO group than in the non-CRO group (63.4% vs 45.9%). Moreover, the CRO group had higher temperature-max and SOFA score, whereas lower hemoglobin-min. Regarding treatment measures, the CRO group had greater use of ventilators, peripherally inserted central catheter (PICC)-line, arterial-line, dialysis-line, tracheotomy, and RRT. Finally, the CRO group had a higher frequency of using cephalosporins and carbapenems. After PSM, the two cohorts showed no significant difference (P>0.05) and were comparable (**Table S1**).

**Table 1:** The characteristics of included patients when the first ICU admission.

Variables	All patients (n=4531)	Non-CRO patients (n=4348)	CRO patients (n=183)	*P*
Male, n (%)	2112 (46.6)	1996 (45.9)	116 (63.4)	<0.001
Age, years	70.00 (58.00, 80.00)	70.00 (59.00, 81.00)	67.00 (54.00, 78.00)	
Weight	76.00 (63.60, 92.50)	76.00 (63.60, 92.40)	76.80 (64.50, 94.95)	
Vital signs^a^				
MAP_min, (mmHg)	57.00 (51.00, 65.00)	57.75 (51.00, 65.00)	56.00 (49.75, 63.00)	
Temperature_max, (°C)	37.61 (37.05, 38.44)	37.55 (37.05, 38.44)	38.02 (37.27, 38.86)	<0.001
Heartrate_max, (min^−1^)	108.00 (94.00, 123.00)	108.00 (93.00, 123.00)	112.00 (102.00, 127.00)	
SpO2_min,(%)	92.00 (89.00, 94.00)	92.00 (89.00, 94.00)	92.00 (90.00, 94.00)	
Severity Score				
SOFA	4.00 (3.00, 7.00)	4.00 (2.00, 7.00)	5.00 (3.00, 8.00)	<0.001
SAPS II	30.00 (23.00, 39.00)	30.00 (23.00, 39.00)	33.00 (24.00, 41.00)	
Comorbidity, n(%)				
Diabetes, n(%)	1580 (34.9)	1518 (34.9)	62 (33.9)	
Liver disease, n (%)	782 (17.3)	746 (17.2)	36 (19.7)	
COPD, n(%)	393 (8.7)	376 (8.6)	17 (9.3)	
Malignant cancer, n(%)	875 (19.3)	839 (19.3)	36 (19.7)	
Cerebrovascular disease, n(%)	1114 (24.6)	1081 (24.9)	33 (18.0)	
Hypoimmunity, n(%)	976 (21.5)	937 (21.6)	39 (21.3)	
Laboratory tests^b^				
Glucose_max, (mg/dl)	146.00 (119.00, 186.00)	146.00 (119.00, 186.00)	151.50 (121.25, 184.00)	
BUN_max, (mg/dL)	24.00 (16.00, 39.00)	24.00 (16.00, 38.00)	29.00 (16.25, 43.00)	
Creatinine-max, (μmol/L)	1.00 (0.70, 1.60)	1.00 (0.70, 1.60)	1.00 (0.70, 1.70)	
Hemoglobin_min, (g/dL)	9.30 (8.00, 10.70)	9.30 (8.10, 10.80)	8.40 (7.70, 9.40)	<0.001
WBC_max, (K/uL)	12.70 (9.10, 17.10)	12.60 (9.10, 17.10)	13.65 (8.62, 18.95)	
Platelet_min, (K/uL)	188.00 (126.00, 262.00)	187.00 (126.00, 260.00)	204.00 (130.75, 315.00)	
Pt_max, (s)	14.30 (12.70, 17.50)	14.20 (12.60, 17.50)	15.10 (13.50, 17.80)	
Ptt_max, (s)	32.90 (28.40, 45.95)	32.90 (28.40, 46.35)	32.95 (28.50, 42.72)	
Treatment measures				
Ventilation	1655 (36.5)	1547 (35.6)	108 (59.0)	<0.001
PICC_line	573 (12.6)	522 (12.0)	51 (27.9)	<0.001
Arterial_line	1657 (36.6)	1565 (36.0)	92 (50.3)	<0.001
Dialysis_line	165 (3.6)	138 (3.2)	27 (14.8)	<0.001
Tracheotomy	871 (19.2)	813 (18.7)	58 (31.7)	<0.001
Catheter	1204 (26.6)	1154 (26.5)	50 (27.3)	
CVC	1086 (24.0)	1024 (23.6)	62 (33.9)	
Gastric_tube	2793 (61.6)	2693 (61.9)	100 (54.6)	
RRT	233 (5.1)	204 (4.7)	29 (15.8)	<0.001
Chemotherapy	106 (2.3)	99 (2.3)	7 (3.8)	
Bronchoscopy	189 (4.2)	174 (4.0)	15 (8.2)	
Antimicrobial				
Cephalosporins, n(%)	1157 (25.5)	1082 (24.9)	75 (41.0)	<0.001
Carbapenems, n(%)	145 (3.2)	99 (2.3)	46 (25.1)	<0.001

Categorical data were presented as frequency (percentage), parametric continuous data were presented as median (interquartile ranges), whereas non-parametric continuous data were presented as median (interquartile ranges); COPD, Chronic obstructive pulmonary disease, CVC, Central venous catheter.

a Vital signs were calculated during the first 24 h since ICU admission of each included patients.

b The laboratory tests recorded the worst value during the first 24 h since ICU admission of each included patients.

### CRO Infection Was Independently Associated With ICU-LOS and Mortality

**Figure 2:**
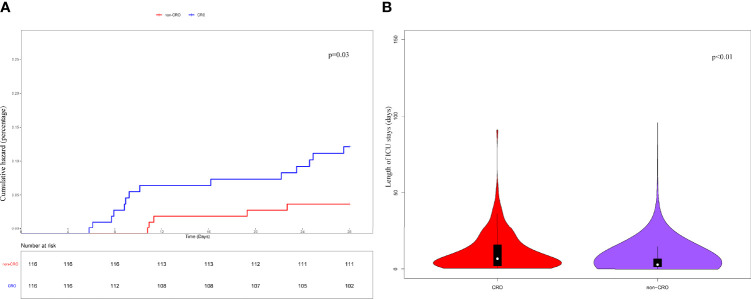
Kaplan-Meier’s survival estimated of the 28-day **(A)** survival probability of CRO and non-CRO patients. The results showed that the 28-day survival of CRO patients was significantly lower than that of non-CRO patients (P<0.05). And the ICU-LOS **(B)** of CRO patients was longer than non-CRO patients (P<0.01).

Subsequently, a PSM was conducted between the CRO and non-CRO cohorts according to the differences in sex, laboratory tests, SOFA score, treatment measures, and use of antimicrobials to estimate the impact of CRO infection on 28-day survival and ICU-LOS. The CRO and non-CRO groups were matched on propensity scores by 1:1 matching with replacement. K-M survival analysis showed significant differences between the CRO and non-CRO groups in 28-day survival (P < 0.05) and ICU-LOS (P < 0.01) (**Figure 2**).

### Development of a Predictive Nomogram and Its Validation

**Figure 3:**
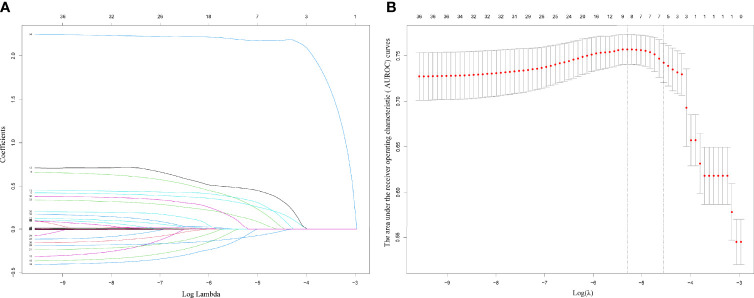
Plots for LASSO regression coefficients over different values of the penalty parameter **(A)**. Cross validation plot for the penalty term **(B)**.

**Figure 4:**
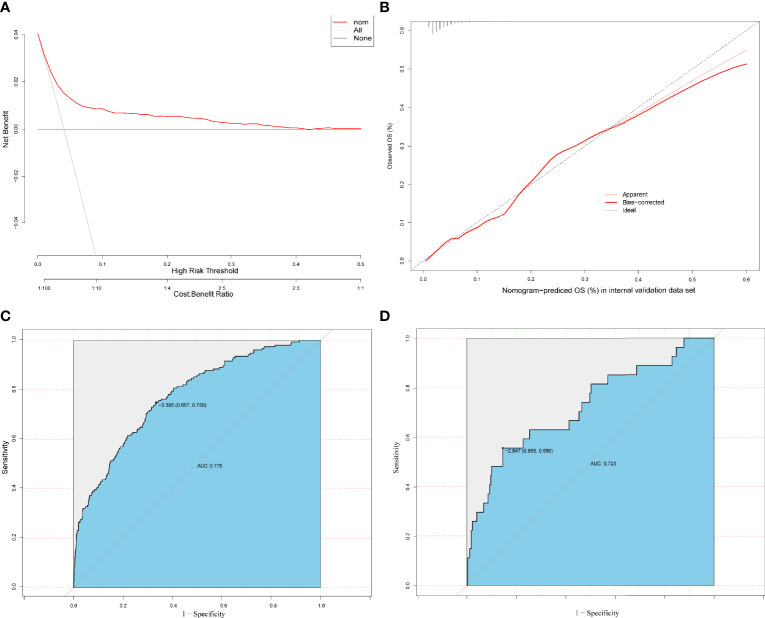
Decision curve analysis (DCA) of the prediction models based on the training and validation set **(A)**. Calibration curves were constructed in the training and validation **(B)** set. The ROC curve of the prediction models is based on training **(C)** or validation **(D)** set.

**Figure 5:**
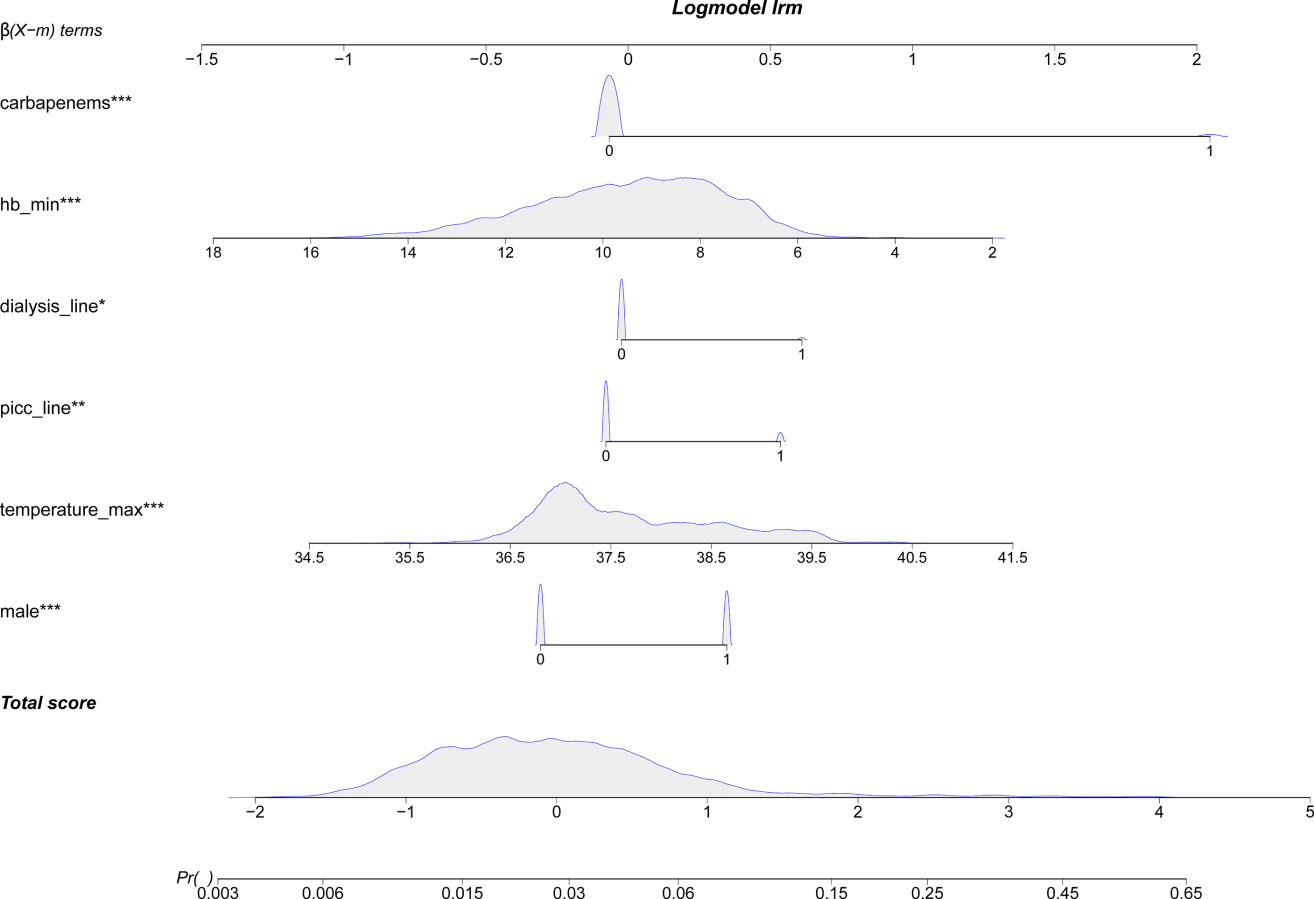
Nomogram to predict the acquisition risk of CRO of patients with the first ICU admission.

The results on the 36 variables included in the LASSO regression and their corresponding regression coefficients are shown in **Figure 3**. When the variables were reduced to six (namely, male sex, hemoglobin [Hb]-min, temperature-max, PICC use, dialysis treatment, and use of carbapenems), the model conferred better performance and was cost-effective. Next, an LRM integrating the six variables was established to predict the CRO acquisition risk using the training set, and the model had a better discrimination ability and performance as indicated by the AUROC curve of 0.776 (95% confidence interval [CI] 0.667-0.750) and 0.723 (95% CI 0.556-0.855) in the training set (**Figure 4C**) and validation (**Figure 4D**) set respectively, and resulting in a sensitivity of 55.56% and a specificity of 85.48%. Then we developed a nomogram to predict CRO infection risk based on the six variables, with an aim toward clinical application (**Figure 5**), and plotted the curve in decision curve analysis (**Figure 4A**) and the calibration curve (**Figure 4B**). The nomogram provided a greater benefit and stability. Finally, we developed a dynamic nomogram for more extensive validation (https://amu-cre-web.shinyapps.io/dynnomapp/).

## Discussion

This study showed that the risk factors for CRO infection were male sex, Hb-min, temperature-max, PICC use, dialysis treatment, and use of carbapenems. The nomogram was established to predict the risk of acquiring CRO infection based on these six variables determined by Logistic regression analysis. The nomogram was also internally validated, wherein a better performance was noted, and because of geographical, racial, and medication differences, external validation was not performed.

When patients are admitted to the ICU for the first time, we may tend to overlook their risk of acquiring CRO infection and may not perform monitoring. Public health workers can use this nomogram to easily assess the risk of acquiring CRO infection among ICU patients and to decide on the development of concrete prevention and control strategies.

In this study, approximately 4.04% of patients with first ICU admission had CRO infection, a proportion that was lower than that reported in other studies (Li et al., 2019; Giannella et al., 2021; Seo et al., 2021). Patients with CRO infection had significantly higher ICU-LOS, treatment costs, and mortality rates (Tansarli et al., 2013; Birgand et al., 2016; Ho et al., 2016; Avendano et al., 2020; Merrick et al., 2021). Early identification of patients with CRO infection is important for disease prevention and control. The prevalence of CRO infection in a region is influenced by multiple factors (Wilson, 2016) such as the overall medical care level, age, administration of antibiotics, disease severity, and so on. Moreover, CRO in this study was defined based on database data, which may have had many influences on the true infection rate.

CRO mainly include CRE, CRAB, and CRPA. Currently available surveillance and screening measures primarily target CRE (Gniadek et al., 2016), which is an essential component of CRO. In the US, it is reported that the common risk factors for CRE infection are the underlying disease severity, long-term hospital admissions, indwelling invasive devices, poor functional status, exposure to antimicrobials, and increasing colonization pressure (Guh et al., 2014). At a secondary referral hospital in Korea, a retrospective study suggested that the risk factors for carbapenem-resistant infection among ICU patients were indwelling nasogastric tube and central venous catheter (CVC), a high Acute Physiology and Chronic Health Evaluation (APACHE) II score, and administration of cephalosporin antibiotics (Seo et al., 2021). Additional risk factors were reported in a China study (Li et al., 2019). However, the risk of carbapenem resistance in glucose-nonfermenting gram-negative bacilli (CR-NF), such as *Pseudomonas aeruginosa* and *Acinetobacter baumannii*, is increasing significantly (Gniadek et al., 2016). However, the methods for the surveillance and screening of CR-NF are still controversial.

In this study, we sought to investigate the acquisition risk of CRO infection. Interestingly, we found that male sex was associated with the risk of acquiring CRO infection. This may be related to the constitutive ratio of CRO-infected individuals and differences in the immunity between sexes (Klein et al., 2015). In a tertiary teaching hospital in China, it is reported that 80% patients with CRE infection were male, and male sex is an independent risk factor for CRE infection (Liu, 2016). And in ICU, compared with female patients, male patients were more likely to undergo invasive treatments, such as deep venous puncture, mechanical ventilation and dialysis (Xu et al., 2019), which may increase the risk of CRO infection. Further studies are needed to investigate the relationship between sex and the risk of developing CRO infection. Anemia and fever are important characteristic features of CRO infection. Anemia is frequently seen in different forms of the infection (Kwaan, 2011), which may be related to abnormalities in iron metabolism (Jonker and Boele van Hensbroek, 2014). PICC refers to catheters placed into the central vein by peripheral venipuncture. A meta-analysis showed that PICC-related central line-associated bloodstream infection (CLABSI) occurred as frequently as CVC-related CLABSI (Chopra et al., 2013). As per our understanding, indwelling PICC is often used for specific categories of patients or for patients requiring prolonged use, which will lead to an increased risk of CRO infection. Dialysis is one of the important supportive measures for ICU patients. The latest matched case-control study suggested that ICU patients undergoing dialysis may be at increased risk for CRE infection, a finding that is consistent with those reported in a study (Aleidan et al., 2021). The same may be true for the risk of CRO infection. Inappropriate use of antimicrobials is an important reason for the development of bacterial resistance. Studies have suggested a positive association between exposure to broad-spectrum antimicrobials and carbapenem resistance (Sampaio and Gales, 2016; Moawad et al., 2018; Yang et al., 2018; Alzomor et al., 2019). Strict management of antimicrobial use remains the cornerstone to avoid bacterial resistance.

Currently, multiple risk factors are strongly associated with CRO infection. However, there is established no scoring system available to predict the risk of CRO infection. Partly, the reasons may be the differences in medical conditions, economic development levels, and antimicrobial usage in different regions (Liu et al., 2015). In this study, we developed a nomogram to predict CRO acquisition risk in patients with first ICU admission, which showed better performance. Clinicians can dynamically assess each patient’s CRO acquisition risk using the developed nomogram. If the score increases significantly, then we should make more efforts to control the risk factors and conduct targeted surveillance. For example, if a male patient in an ICU presents with fever and anemia, has the use of carbapenems and an implanted catheter, and undergoes dialysis, then we should actively manage the fever and anemia; reassess the need for catheters, dialysis, and carbapenems; and complete active screening for infection. By controlling for the emerging risk factors, the occurrence of CRO infection could be decreased. Meanwhile, we can identify patients with CRO infection at an early stage and reduce nosocomial transmission through targeted active surveillance. Accordingly, costs associated with the prevention and control of the infection will also decrease.

This study had several limitations. First, the incidence of CRO infection was calculated based on culture-positive patients in the MIMIC-IV database. It is possible that a few patients with CRO infection remained undiagnosed. Second, because of geographical variations, risk factors for CRO infection may not have been entirely consistent. Finally, the newly established nomogram was not validated based on external data. And the sensitivity of the nomogram was medium (0.55), the specificity was good (0.85), the negative predictive value was high (0.98), but the positive predictive value was not high (0.14) (**Table S2**). It may be related to the sample size and the prevalence of CRO infection. As a complementary measure, we plotted a dynamic nomogram for more extensive validation.

In conclusion, a nomogram that included six variables was developed to predict CRO acquisition risk in ICU patients based on a LASSO regression and Logistic analysis, which showed a good performance through internal validation. Based on scoring using the nomogram, we can aim to control the risk factors and conduct targeted active surveillance in a timely manner for the purpose of reducing the occurrence of CRO infection, identifying patients with CRO infection at an early stage, reducing nosocomial transmission, and decreasing costs related to prevention and control. Further external validation is needed to optimize and improve the nomogram.

## Data Availability Statement

The datasets presented in this study can be found in online repositories. The names of the repository/repositories and accession number(s) can be found in the article/**Supplementary Material**.

## Ethics Statement

Ethical review and approval was not required for the study on human participants in accordance with the local legislation and institutional requirements. Written informed consent for participation was not required for this study in accordance with the national legislation and the institutional requirements.

## Author Contributions

MY and JZ: concept. JZ and WL: methodology and writing of the manuscript. WS and XC: data processing. WX and YL: software. MY and TH: review and editing. All authors contributed to the article and approved the submitted version.

## Funding

This study was supported by a research grant from the National Natural Science Foundation of China (No.82072134) and the Clinical Research cultivation Program of the Second Affiliated Hospital of Anhui Medical University (No.2020LCYB03).

## Conflict of Interest

The authors declare that the research was conducted in the absence of any commercial or financial relationships that could be construed as a potential conflict of interest.

## Publisher’s Note

All claims expressed in this article are solely those of the authors and do not necessarily represent those of their affiliated organizations, or those of the publisher, the editors and the reviewers. Any product that may be evaluated in this article, or claim that may be made by its manufacturer, is not guaranteed or endorsed by the publisher.

## Supplementary Material

Supplementary Figure 1 The distribution of the missing vale.
