# Supplementary material for: A Nomogram With Six Variables Is Useful to Predict the Risk of Acquiring Carbapenem-Resistant Microorganism Infection in ICU Patients
Source: Front Cell Infect Microbiol. 2022 Mar 25;12:852761. doi: 10.3389/fcimb.2022.852761 (PMC8990894; doi:10.3389/fcimb.2022.852761)
Supplement: Supplementary file 2 [file Table_1.doc]

Table S1 The characteristics of included patients when ICU admission after PSM

| Variables | All patients (n=232) | Non-CRO patients (n=116) | CRO patients (n=116) | *P* |
| --- | --- | --- | --- | --- |
| Male, n (%) | 141 (40.8) | 76 (65.5) | 65 (56.0) | >0.05 |
| Age, years | 67.50 (57.00, 77.25) | 67.50 (57.00, 75.00) | 67.50 (55.50, 79.00) | >0.05 |
| Weight | 75.80 (64.35, 94.43) | 79.40 (65.27, 93.12) | 71.75 (61.30, 94.93) | >0.05 |
| Vital signs^a^ |  |  |  |  |
| MAP_min, (mmHg) | 57.00 (50.00, 65.00) | 57.50 (50.75, 67.00) | 57.00 (50.00, 63.00) | >0.05 |
| Temperature_max, (℃) | 37.83 (37.27, 38.66) | 37.83 (37.27, 38.61) | 37.83 (37.27, 38.70) | >0.05 |
| Heartrate_max, (min^−1^) | 111.00 (94.75, 124.00) | 114.00 (94.00, 127.00) | 108.50 (97.50, 121.00) | >0.05 |
| SpO2_min,(%) | 92.00 (90.00, 95.00) | 92.00 (89.00, 95.00) | 92.00 (90.00, 95.00) | >0.05 |
| Severity Score |  |  |  |  |
| SOFA | 5.00 (3.00, 7.00) | 5.00 (3.00, 7.00) | 5.00 (3.00, 7.00) | >0.05 |
| SAPS II | 31.00 (24.00, 40.00) | 30.00 (24.00, 39.00) | 32.00 (24.00, 40.00) | >0.05 |
| Comorbidity, n(%) |  |  |  |  |
| Diabetes, n(%) | 79 (34.1) | 40 (34.5) | 39 (33.6) | >0.05 |
| Liver disease, n (%) | 44 (19.0) | 20 (17.2) | 24 (20.7) | >0.05 |
| COPD, n(%) | 17 (7.3) | 7 (6.0) | 10 (8.6) | >0.05 |
| Malignant cancer, n(%) | 43 (18.5) | 23 (19.8) | 20 (17.2) | >0.05 |
| Cerebrovascular disease, n(%) | 42(18.1) | 20 (17.2) | 22 (19.0) | >0.05 |
| Hypoimmunity, n(%) | 49 (21.1) | 26 (22.4) | 23 (19.8) | >0.05 |
| Laboratory tests^b^ |  |  |  |  |
| Glucose_max, (mg/dl) | 150.50 (120.00, 188.00) | 147.00 (120.00, 182.75) | 160.00 (121.25, 192.00) | >0.05 |
| BUN_max, (mg/dL) | 25.50 (16.00, 39.00) | 25.00 (16.00, 37.25) | 26.00 (16.75, 42.00) | >0.05 |
| Creatinine-max, (μmol/L) | 1.00 (0.70, 1.60) | 1.10 (0.80, 1.60) | 0.90 (0.60, 1.60) | >0.05 |
| Hemoglobin_min, (g/dL) | 8.55 (7.80, 9.90) | 8.60 (7.90, 10.07) | 8.50 (7.80, 9.75) | >0.05 |
| WBC_max, (K/uL) | 12.60 (8.30, 17.92) | 12.20 (8.73, 17.28) | 13.05 (8.07, 18.25) | >0.05 |
| Platelet_min, (K/uL) | 186.50 (129.50, 271.25) | 179.00 (124.25, 240.25) | 204.00 (141.50, 308.50) | >0.05 |
| Pt_max, (s) | 14.30 (12.80, 16.35) | 14.10 (12.62, 16.10) | 14.50 (13.10, 17.00) | >0.05 |
| Ptt_max, (s) | 32.00 (27.98, 40.90) | 31.75 (27.80, 40.90) | 32.55 (28.25, 40.95) | >0.05 |
| Treatment measures |  |  |  |  |
| Ventilation | 111 (47.8) | 54 (46.6) | 57 (49.1) | >0.05 |
| PICC_line | 39 (16.8) | 18 (15.5) | 21 (18.1) | >0.05 |
| Arterial_line | 98 (42.2) | 48 (41.4) | 50 (43.1) | >0.05 |
| Dialysis_line | 14 (6.0) | 7 (6.0) | 7 (6.0) | >0.05 |
| Tracheotomy | 57 (24.6) | 24 (20.7) | 33 (28.4) | >0.05 |
| Catheter | 54 (23.3) | 29 (25.0) | 25 (21.6) | >0.05 |
| CVC | 60 (25.9) | 25 (21.6) | 35 (30.2) | >0.05 |
| Gastric_tube | 137 (59.1) | 70 (60.3) | 67 (57.8) | >0.05 |
| RRT | 16 (6.9) | 7 (6.0) | 9 (7.8) | >0.05 |
| Chemotherapy | 8 (3.4) | 5 (4.3) | 3 (2.6) | >0.05 |
| Bronchoscopy | 17 (7.3) | 8 (6.9) | 9 (7.8) | >0.05 |
| Antimicrobial |  |  |  |  |
| Cephalosporins, n(%) | 73 (31.5) | 35 (30.2) | 38 (32.8) | >0.05 |
| Carbapenems, n(%) | 9 (3.9) | 5 (4.3) | 4 (3.4) | >0.05 |

Categorical data were presented as frequency (percentage), parametric continuous data were presented as median (interquartile ranges), whereas non-parametric continuous data were presented as median (interquartile ranges); COPD Chronic obstructive pulmonary disease, CVC Central venous catheter;

^a^Vital signs were calculated during the first 24 h since ICU admission of each included patients;

^b^The laboratory tests recorded the worest value during the first 24 h since ICU admission of each included patients.
