# Supplementary material for: A Nomogram With Six Variables Is Useful to Predict the Risk of Acquiring Carbapenem-Resistant Microorganism Infection in ICU Patients
Source: Front Cell Infect Microbiol. 2022 Mar 25;12:852761. doi: 10.3389/fcimb.2022.852761 (PMC8990894; doi:10.3389/fcimb.2022.852761)
Supplement: Supplementary file 3 [file Table_2.doc]

Table S2 The predictive performance of nomogram

| Accuracy (95% CI) | Sensitivity (95% CI) | Specificity (95% CI) | PPV (95% CI) | NPV(95% CI) |
| --- | --- | --- | --- | --- |
| 0.84(0.81-0.87) | 0.55(0.35-0.75) | 0.85(0.82-0.88) | 0.14(0.10-0.19) | 0.98(0.97-0.99) |

CI: confidence interval; PPV: positive predictive value; NPV: negative predictive value.
